# Outcomes of Retrograde Intrarenal Surgery Performed Under Neuraxial vs. General Anesthesia: An Updated Systematic Review and Meta-Analysis

**DOI:** 10.3389/fsurg.2022.853875

**Published:** 2022-03-10

**Authors:** Mingda Duan, Yu Chen, Li Sun

**Affiliations:** ^1^Department of Anesthesiology, Hainan Hospital of Chinese PLA General Hospital, Sanya, China; ^2^Department of Anesthesiology, Sixth Medical Center of Chinese PLA General Hospital, Beijing, China; ^3^Department of Anesthesiology, First Medical Center of Chinese PLA General Hospital, Beijing, China

**Keywords:** urolithiasis, nephrolithiasis, ureterorenoscopy, kidney stone, anesthesia

## Abstract

**Background:**

The current review aimed to assess if the outcomes of retrograde intrarenal surgery (RIRS) differ with neuraxial anesthesia (NA) or general anesthesia (GA).

**Methods:**

The databases of PubMed, Embase, CENTRAL, ScienceDirect, and Google Scholar were searched up to 3rd December 2021 for randomized controlled trials (RCTs) and observational studies comparing outcomes of RIRS with NA or GA.

**Results:**

Thirteen studies involving 2912 patients were included. Eight were RCTs while remaining were observational studies. Meta-analysis revealed that stone free status after RIRS did not differ with NA or GA (OR: 0.99 95% CI: 0.77, 1.26 I^2^ = 10% *p* = 0.91). Similarly, there was no difference in operation time (MD: −0.35 95% CI: −4.04, 3.34 I^2^ = 89% *p* = 0.85), 24 h pain scores (MD: −0.36 95% CI: −0.96, 0.23 I^2^ = 95% *p* = 0.23), length of hospital stay (MD: 0.01 95% CI: −0.06, 0.08 I^2^ = 35% *p* = 0.78), Clavien-Dindo grade I (OR: 0.74 95% CI: 0.52, 1.06 I^2^ = 13% *p* = 0.10), grade II (OR: 0.70 95% CI: 0.45, 1.07 I^2^ = 0% *p* = 0.10) and grade III/IV complication rates (OR: 0.78 95% CI: 0.45, 1.35 I^2^ = 0% *p* = 0.37) between NA and GA. Except for grade I complications, the results did not change on subgroup analysis based on study type and NA type.

**Conclusion:**

Our results suggest that NA can be an alternative to GA for RIRS. There seem to be no difference in the stone-free rates, operation time, 24-h pain scores, complication rates, and length of hospital stay between NA and GA for RIRS. Considering the economic benefits, the use of NA may be preferred over GA while taking into account patient willingness, baseline patient characteristics, and stone burden.

**Systematic Review Registration:**

https://www.crd.york.ac.uk/prospero/, identifier: CRD42021295407.

## Introduction

Urolithiasis is a highly prevalent urological disease that significantly impacts the health and quality of life of affected individuals (1). The prevalence of the disease varies across different regions in the world, ranging from 0.1 to 14.8% in Western countries to up to 10.6% in the Asian population (1, 2). Furthermore, 50% of individuals with urolithiasis suffer from recurrence within 5 to 10 years of first diagnosis (3). The presence of urolithiasis can cause significant morbidity in an individual leading to symptoms like infection, flank pain, hydronephrosis, and decreased renal function (4). Indeed, colic pain due to renal stone is a very common presentation in an emergency department (5).

Several treatment methods are available for managing urolithiasis ranging from observation, medical expulsive therapy, shockwave lithotripsy, percutaneous nephrolithotomy (PCNL), retrograde intrarenal surgery (RIRS), to laparoscopic or open renal surgeries for extreme cases (3). Of the minimally-invasive treatment modalities, RIRS has superior stone-free rates as compared to shockwave lithotripsy and reduced complications as compared to PCNL, which makes it an attractive treatment option (6, 7). Recent guidelines suggest that RIRS is a safe and effective treatment modality for stones up to 20 mm in size (8, 9). Furthermore, advances in technology and equipment like the development of new laser systems, flexible ureteroscopy with miniaturized ureteroscopes have not only expanded the indications of RIRS but also have made the procedure safer with a short duration of hospitalization and low rate of complications (10).

However, as RIRS has been traditionally performed under general anesthesia (GA), several anesthetic complications still occur which can add to the complexity as well as the cost of the procedure. In this context, the role of regional anesthesia, specifically neuraxial anesthesia (NA) has been explored for RIRS in recent times. NA can be advantageous as compared to GA as it eliminates several GA-related adverse events and allows for early mobilization (11). Over the past decade, several studies have compared outcomes of NA vs. GA for RIRS but with variable results. To the best of our knowledge, only two systematic reviews (12, 13) have been attempted to pool evidence on the impact of anesthesia on RIRS outcomes. However, a major limitation of these reviews was that only six studies could be included in the analysis. Considering the publication of new literature, there is a strong need for updated evidence. Thus, the current review aimed to pool evidence from the literature to compare the efficacy and safety of RIRS performed under NA vs. GA.

## Materials and Methods

The reporting guidelines of the PRISMA statement (Preferred Reporting Items for Systematic Reviews and Meta-analyses) (14) and the Cochrane Handbook for Systematic Reviews of Intervention (15) were followed for this review. We registered the study protocol prospectively on PROSPERO (No CRD42021295407). The protocol was registered to compare regional anesthesia with GA for RIRS. However, since the majority of the studies were on NA and only one study used peripheral nerve block, we modified the review to compare outcomes of NA vs. GA for RIRS.

### Database Search

An electronic literature search was conducted on the databases of PubMed, Embase, CENTRAL, and ScienceDirect. Gray literature was searched using Google Scholar, only for the first 100 results for each query. We sought the aid of a medical librarian to formalize the search strategy. The databases were searched by two reviewers separately while defining the search limits from the inception of the databases to 3rd December 2021. No language restriction was applied. A combination of MeSH and free-text keywords were, namely: “retrograde intrarenal surgery,” “RIRS,” “ureterorenoscopy,” “ureterolithotripsy,” “FURS,” and “anesthesia.” Details of the literature search common to all databases are presented in Supplementary Table 1. The primary search results were assessed initially by their titles and abstracts to identify citations requiring full-text analysis. The full texts of the articles were reviewed by the two reviewers independently based on the inclusion and exclusion criteria. All disagreements were resolved in consultation with the third reviewer. We also cross-checked the references of included studies and prior systematic reviews on the topic to look for any additional articles.

### Inclusion Criteria

Inclusion criteria were defined as per the PICOS (Population, Intervention, Comparison, Outcome, and Study design) framework, which included:

Population: Adult patients (>18 years of age) with urolithiasis undergoing RIRS.
Intervention: NA including spinal or epidural or combined spinal-epidural.
Comparison: GA.
Outcomes: Stone-free rates, and/or operation time, and/or pain scores, and/or complications.
Study design: Randomized controlled trials (RCTs), controlled clinical trials, and observational studies

Exclusion criteria were: 1) non-comparative studies 2) Studies combining regional anesthesia with GA 3) Studies not reporting relevant outcomes 4) Studies published only as abstracts.

### Data Extraction and Risk of Bias Assessment

A data extraction sheet was used by two reviewers to extract relevant data from the studies. Details of the first author, publication year, study location, surgery type, sample size, demographic details, mean stone size and density, stone side (right/left), the definition of stone free status, study outcomes, follow-up duration, and imaging used on follow-up were extracted. The primary outcome of interest was stone-free status. We did not pre-define this outcome and used the definition of the included studies. Secondary outcomes were operation time, postoperative pain scores, complications graded by Clavien-Dindo classification, and length of hospital stay (LOS).

The included RCTs were assessed for risk of bias using the Cochrane Collaboration's risk of bias assessment tool-2 (15). The domains evaluated were: randomization process, deviation from intended intervention, missing outcome data, measurement of outcomes, and selection of reported results. Based on the risk of bias in individual domains, the overall bias was marked as “high risk,” “some concerns,” or “low risk.” For non-RCTs, the risk of a bias assessment tool for non-randomized studies (RoBANS) was used (16). Studies were assessed for: selection of participants, confounding variables, intervention measurements, blinding of outcome assessment, incomplete outcome data, and selective outcome reporting. The quality assessment was carried out by two reviewers separately and any disagreements were resolved in consultation with the third reviewer. The certainty of the evidence of only RCTs was assessed using the Grading of Recommendations Assessment, Development, and Evaluation (GRADE) tool using the GRADEpro GDT software [GRADEpro Guideline Development Tool. McMaster University, 2020 (developed by Evidence Prime, Inc.)].

### Statistical Analysis

We used “Review Manager” (RevMan, version 5.3; Nordic Cochrane Centre [Cochrane Collaboration], Copenhagen, Denmark; 2014) for the meta-analysis. Continuous outcomes like operation time and pain scores were pooled using the mean difference (MD) and 95% confidence intervals (CI). Dichotomous data like stone-free status, complications were pooled using odds ratios (OR). The random-effects model was used for all the meta-analyses. Heterogeneity was assessed using the I^2^ statistic. I^2^ values of 25–50% represented low, values of 50–75% medium, and more than 75% represented substantial heterogeneity. Publication bias was assessed using visual inspection of funnel plots. We also conducted a sensitivity analysis by excluding one study at a time in the meta-analysis software itself to look for any change in the significance of the results. Sub-group analyses were conducted based on study type and type of NA.

## Results

### Details of Included Studies

**Figure 1:**
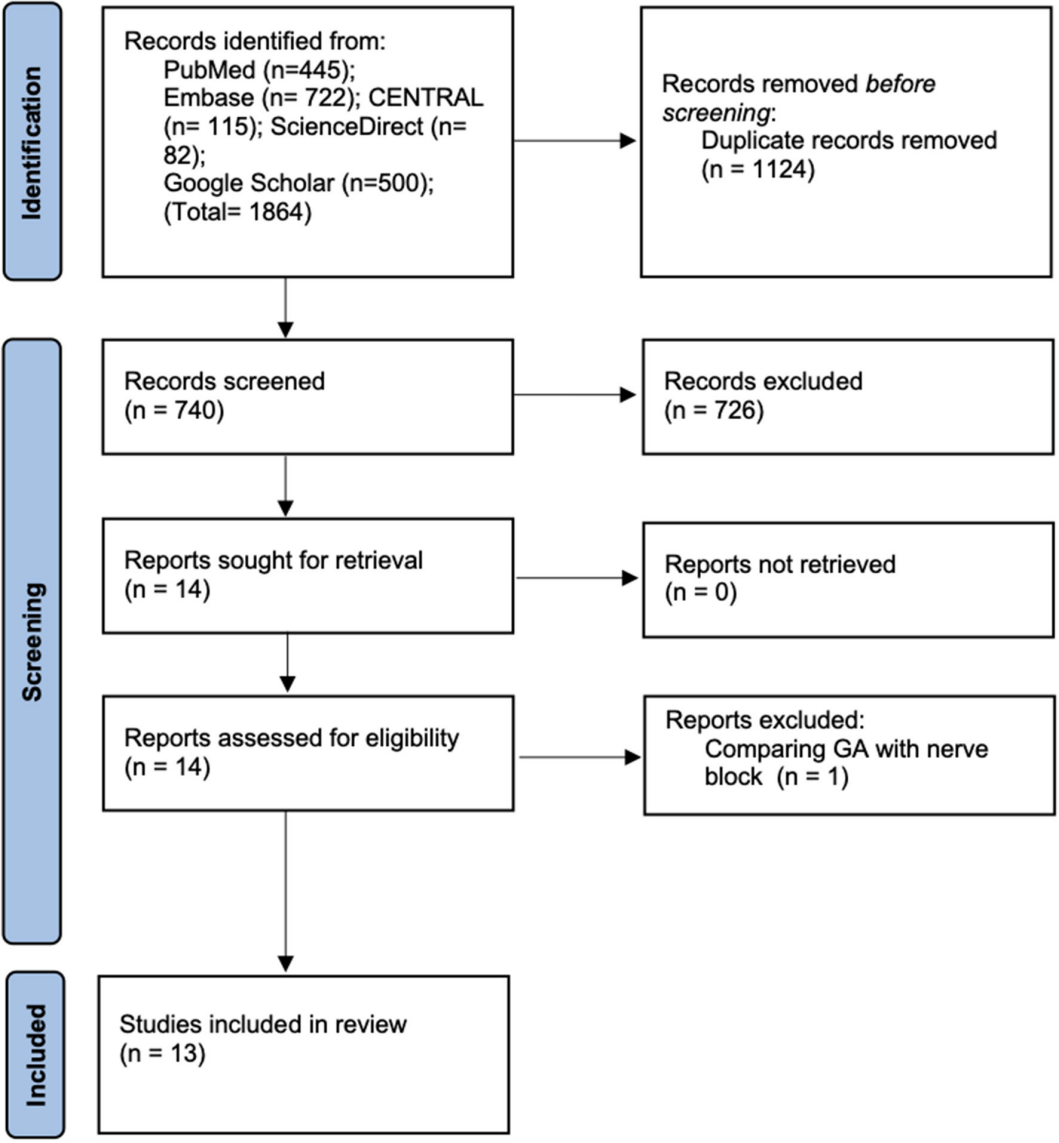
Study flow chart.

Details of the literature search at every stage are presented in Figure 1. A total of 1,864 articles were found on initial screening. Deduplication revealed a total of 740 records of which 726 were excluded based on title and abstract screening. Fourteen articles were retrieved for full-text analysis of which one was excluded as it compared peripheral nerve block with GA. Finally, a total of 13 studies were included in this systematic review and meta-analysis (17–29).

Characteristics of included studies are presented in Table 1. Eight studies were RCTs while the remaining were prospective or retrospective cohort studies. Four studies compared combined epidural and spinal anesthesia with GA while seven studies compared only spinal anesthesia with GA. Two studies (19, 25) conducted a three-arm analysis comparing epidural vs. spinal vs. GA. For the meta-analysis, we combined data of epidural and spinal groups of these two studies into a single group. However, for subgroup analysis based on NA type, we used data of spinal anesthesia from these studies. A total of 1,567 patients receiving NA were compared with 1,345 GA patients in the included studies. The definition of stone-free status varied across the included studies with the minimally acceptable stone size cut-off ranging from 1 to 4 mm. The follow-up duration ranged from 2 weeks to 3 months. Imaging type on follow-up also varied with studies using radiographs, ultrasound, and/or computed tomography.

**Table 1:** Details of included studies.

**References**	**Country**	**Study design**	**Groups**	**Sample size**	**Mean age (years)**	**Gender M/F**	**Mean BMI Kg/m^**2**^**	**Mean stone size (mm)**	**Mean stone density (HU)**	**Stone side R/L**	**Cut-off size for stone free status (mm)**	**Follow-up duration (months)**	**Follow up imaging**
Li et al. (23)	China	RCT	CSEA GA	89 105	31.2 ± 9 36.3 ± 7.9	39/50 63/42	NR	NA	NR	NR	≤ 3	NR	NR
Zeng et al. (29)	China	RCT	CSEA GA	31 34	47.6 ± 11.6 49.3 ± 11.6	20/11 20/14	23.3 ± 2.9 23.4 ± 3.9	19 ± 9 24 ± 13	847.6 ± 295.2 811.8 ± 294.7	19/12 20/14	≤ 3	1	CT
Bosio et al. (18)	Italy	RC	SA GA	139 47	52 ± 14.8 48.7 ± 12.8	80/59 29/18	NR	14 ± 6 14 ± 4.9	NR	NR	≤ 4	0.5	KUB, USG
Karabulut et al. (21)	Turkey	RCT	SA GA	43 43	NR	NR	NR	NR	NR	NR	<4	1	KUB, USG, CT
Baran et al. (17)	Turkey	RC	SA GA	697 664	47 ± 14.2 48.4 ± 14	479/218 434/230	26.5 ± 4.3 27.1 ± 4.5	17.6 ± 5.9 17.2 ± 6	779.9 ± 175.9 764 ± 164.9	NR	≤ 2	1	CT
Çakici et al. (20)	Turkey	RCT	CSEA GA	45 50	46.7 ± 14.6 42.8 ± 11.4	26/19 31/19	26.8 ± 3.8 25.4 ± 3.2	16.1 ± 5.3 13.9 ± 7	764 ± 164.9 835.6 ± 189.1	23/22 27/23	<2	1–3	CT
Kwon et al. (22)	South Korea	RCT	SA GA	31 39	54.7 ± 14 54.1 ± 14.5	20/11 26/13	25 ± 3.7 24.7 ± 2.7	12 ± 3.4 11.3 ± 3.3	961.1 ± 354.4 937 ± 294.2	15/16 15/24	<2	2–3	KUB or CT
Oztekin et al. (25)	Turkey	RCT	EA SA GA	35 35 35	47.3 ± 14.8 45.8 ± 15.4 44.9 ± 14.6	22/13 25/10 23/12	23.3 ± 7.1 37.4 ± 61.5 33.4 ± 22.9	11.8 ± 2.9 12.7 ± 3.6 13 ± 3.8	854.6 ± 384.2 1035.8 ± 371.8 1116 ± 294.9	17/18 20/15 20/15	<3	1	CT
Pelit et al. (26)	Turkey	RCT	SA GA	50 50	45.1 ± 14.9 48.9 ± 15.9	32/18 22/28	NR	18.1 ± 5.3 17.3 ± 4.1	NR	19/31 22/28	≤ 3	3	KUB, USG or CT
Sahan et al. (27)	Turkey	RCT	CSEA GA	45 61	44.1 ± 12.6 46 ± 16.3	26/19 35/26	NR	15.7 ± 7.3 17.2 ± 7.7	NR	25/20 30/31	<2	1	CT
Topaktaş et al. (28)	Turkey	RC	SA GA	40 32	41.9 ± 12.3 40.3 ± 13.3	28/12 22/10	NR	10.1 ± 2.2 11.1 ± 2.1	991.7 ± 404 1093.4 ± 489	NR	≤ 3	1	CT
Cai et al. (19)	China	RC	EA SA GA	116 131 145	47.8 ± 11.3 45 ± 11.8 39.8 ± 8.4	82/34 92/39 114/31	25.9 ± 3.1 25.5 ± 2 25.2 ± 1.9	10.9 ± 1.9 11.1 ± 2.9 11.5 ± 3.5	NR	61/55 69/62 71/74	≤ 3	1	CT
Olivero et al. (24)	Italy	PC	SA GA	40 40	55.8 ± 13.9 54.9 ± 16.9	26/14 28/12	25.4 ± 2.9 25 ± 2.6	12.3 ± 5.4 12.3 ± 4.1	NR	NR	≤ 4	1	KUB or USG

*BMI, body mass index; RCT, randomized controlled trial; PC, prospective cohort; RC, retrospective cohort; CSEA, combined spinal epidural anesthesia; EA, epidural anesthesia; SA, spinal anesthesia; GA, general anesthesia; HU, Hounsfield units; KUB, kidney ureter and bladder radiograph; USG, ultrasonography; CT, computed tomography; NR, not reported; R, right; L, left; M, male; F, female*.

### Primary Outcome

**Figure 2:**
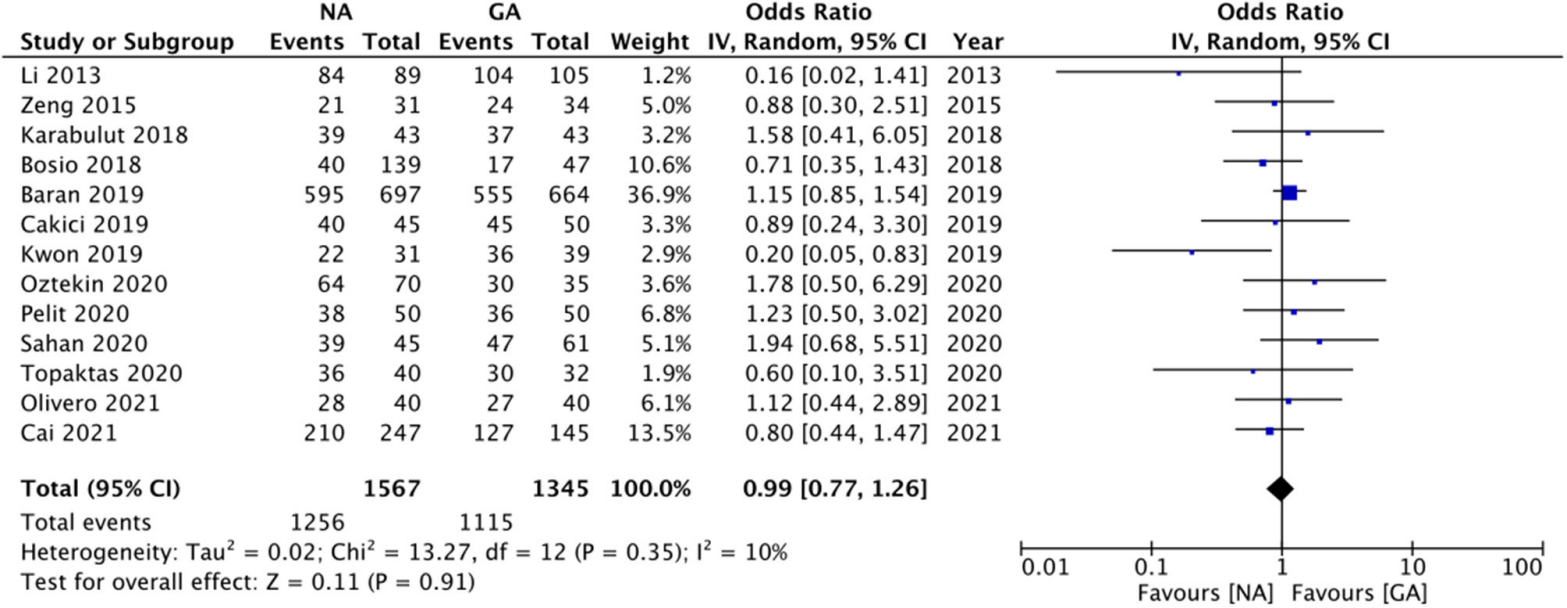
Meta-analysis of stone free rates between NA and GA for RIRS.

All studies reported data on stone-free status. Meta-analysis revealed no statistically significant difference in stone-free status between patients receiving NA or GA (OR: 0.99 95% CI: 0.77, 1.26 I^2^ = 10% *p* = 0.91; Figure 2). There was no evidence of publication bias on visual inspection of the funnel plot (Supplementary Figure 1). On sensitivity analysis, there was no change in the significance of the results on the exclusion of any study. Subgroup analysis based on study type (RCTs or non-RCTs) and NA type (combined epidural and spinal anesthesia or spinal anesthesia only) also did not change the significance of the results (Table 2). The certainty of the evidence of RCTs based on GRADE was “moderate” (Supplementary Table 2).

**Table 2:** Details of subgroup analysis.

**Variable**	**Groups**	**Number of studies**	**Sample size- NA**	**Sample size-GA**	**Effect size**
**Stone free rates**					
Study type	RCTs	8	404	417	OR: 0.97 95% CI: 0.56, 1.65 I^2^ = 34% *p* = 0.90
	Non-RCTs	5	1163	928	OR: 1.01 95% CI: 0.80, 1.29 I^2^ = 0% *p* = 0.91
NA type	CSEA vs. GA	6	527	430	OR: 0.98 95% CI: 0.62, 1.64 I^2^ = 11% *p* = 0.92
	SA vs. GA	9	1206	1095	OR: 0.99 95% CI: 0.76, 1.28 I^2^ = 8% *p* = 0.93
**Operation time**					
Study type	RCTs	7	361	374	MD: 1.33 95% CI: −6.66, 9.31 I^2^ = 89% *p* = 0.74
	Non-RCTs	5	1163	928	MD: −2.95 95% CI: −7.27, 1.36 I^2^ = 88% *p* = 0.18
NA type	CSEA vs. GA	6	527	430	0.40 95% CI: −6.74, 7.55 I^2^ = 92% *p* =0.91
	SA vs. GA	8	1163	1052	MD: −1.43 95% CI: −4.99, 2.13 I^2^ = 82% *p* = 0.43
**Pain 24 h**					
NA type	CSEA vs. GA	4	393	275	MD: −0.36 95% CI: −1.11, 0.38 I^2^ = 96% *p* = 0.34
	SA vs. GA	4	247	269	MD: −0.60 95% CI: −1.30, 0.10 I^2^ = 92% *p* = 0.09
**Complications Grade I**					
Study type	RCTs	5	220	201	OR: 1.73 95% CI: 0.62, 4.82 I^2^ = 0% *p* = 0.29
	Non-RCTs	5	1163	928	OR: 0.67 95% CI: 0.50, 0.88 I^2^ = 0% *p* = 0.004
NA type	CSEA vs. GA	4	393	264	OR: 1.02 95% CI: 0.33, 3.18 I^2^ = 52% *p* = 0.97
	SA vs. GA	8	1136	1045	OR: 0.95 95% CI: 0.69, 1.30 I^2^ = 6% *p* = 0.73
**Complications Grade II**					
Study type	RCTs	4	177	158	OR: 0.44 95% CI: 0.14, 1.35 I^2^ = 0% *p* = 0.15
	Non-RCTs	4	1123	896	OR: 0.68 95% CI: 0.34, 1.36 I^2^ = 34% *p* = 0.28
NA type	CSEA vs. GA	4	393	264	OR: 0.66 95% CI: 0.38, 1.15 I^2^ = 0% *p* = 0.14
	SA vs. GA	6	1073	970	OR: 0.69 95% CI: 0.40, 1.19 I^2^ = 10% *p* = 0.18
**Complications Grade III/IV**					
Study type	RCTs	2	76	84	OR: 1.479 95% CI: 0.21, 14.96 I^2^ = 0% *p* = 0.59
	Non-RCTs	4	1123	888	OR: 0.74 95% CI: 0.44, 1.26 I^2^ = 0% *p* = 0.27
NA type	CSEA vs. GA	3	323	229	OR: 0.93 95% CI: 0.49, 1.75 I^2^ = 0% *p* = 0.81
	SA vs. GA	4	1007	888	OR: 0.73 95% CI: 0.42, 1.29 I^2^ = 0% *p* = 0.28
**Length of hospital stay**					
Study type	RCTs	5	202	234	MD: 0.11 95% CI: −0.06, 0.29 I^2^ = 48% *p* = 0.22
	Non-RCTs	3	327	217	MD: −0.04 95% CI: −0.09, 0.00 I^2^ = 0% *p* = 0.07
NA type	CSEA vs. GA	4	368	290	MD: −0.03 95% CI: −0.07, 0.01 I^2^ = 0% *p* = 0.15
	SA vs. GA	5	292	306	MD: 0.08 95% CI: −0.11, 0.28 I^2^ = 59% *p* = 0.40

*RCTs, randomized controlled trials; CSEA, combined spinal epidural anesthesia; SA, spinal anesthesia; GA, general anesthesia; OR, odds ratio; MD, mean difference*.

### Secondary Outcomes

**Figure 3:**
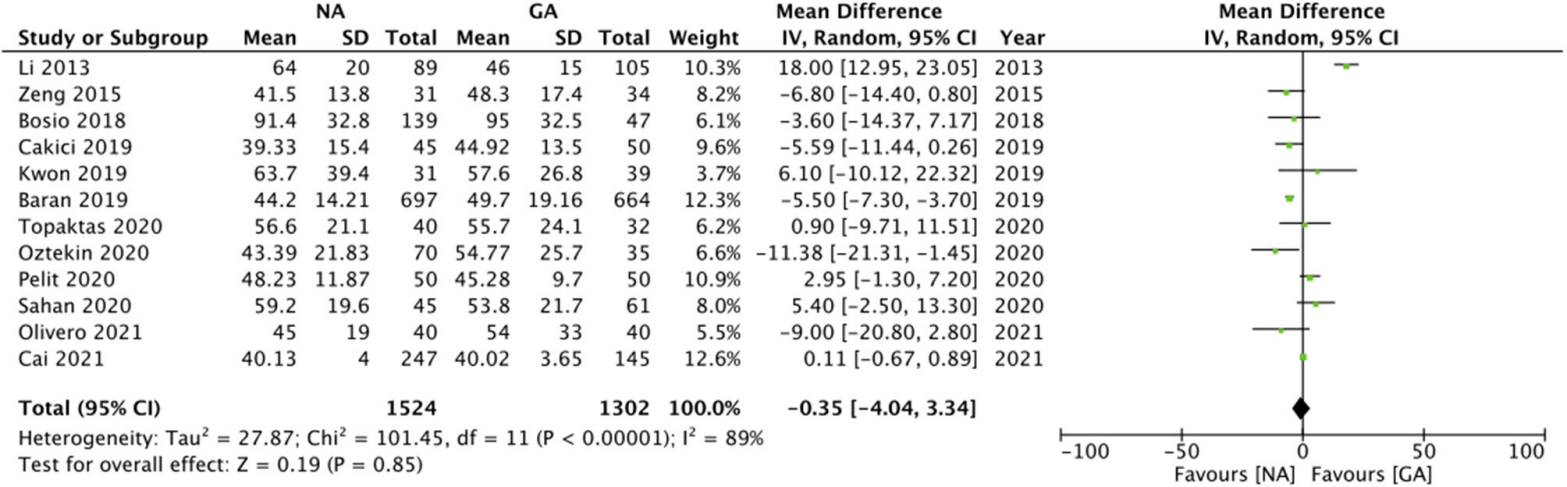
Meta-analysis of operation time between NA and GA for RIRS.

Twelve studies reported data on operation time. Pooled analysis revealed no statistically significant difference in operation time with either anesthesia technique (MD: −0.35 95% CI: −4.04, 3.34 I^2^ = 89% *p* = 0.85; Figure 3). The results did not change in significance on sensitivity analysis. Subgroup analysis based on study type and NA type also demonstrated similar results (Table 2). The certainty of the evidence of RCTs based on GRADE was “moderate” (Supplementary Table 2).

**Figure 4:**
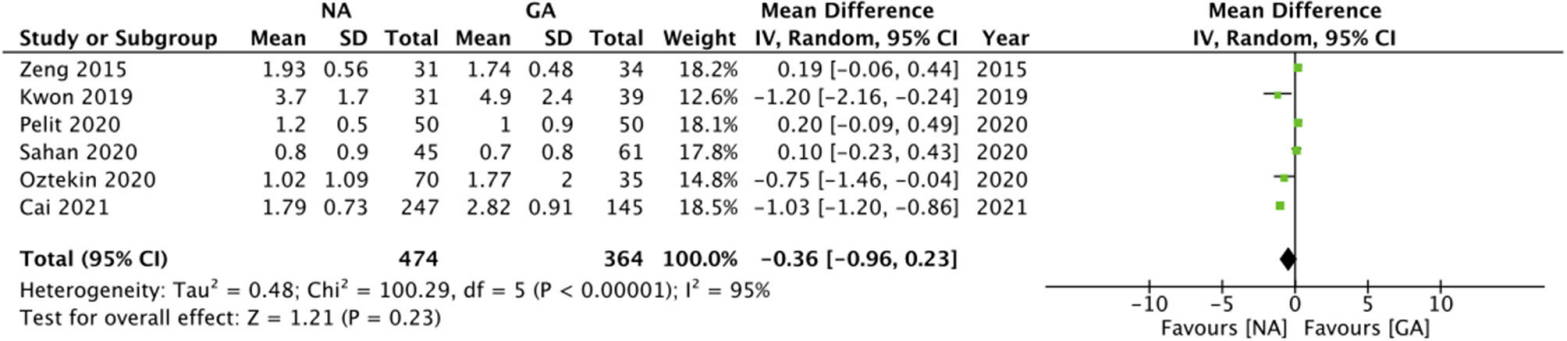
Meta-analysis of 24 h pain scores between NA and GA for RIRS.

The included studies varied significantly in reporting pain outcomes. The only common outcome was pain scores at 24-h which too was reported by only six studies. The meta-analysis demonstrated no statistically significant difference in 24-h pain scores on the Visual analog scale (VAS) in patients undergoing surgery under NA or GA (MD: −0.36 95% CI: −0.96, 0.23 I^2^ = 95% *p* = 0.23; Figure 4). On sensitivity analysis, there was no change in the significance of the results on the exclusion of any study. Since only one of the six studies was an observational study, subgroup analysis based on study type was not conducted. However, subgroup analysis based on NA did not change the significance of the results (Table 2). The certainty of the evidence of RCTs based on GRADE was “moderate” (Supplementary Table 2).

**Figure 5:**
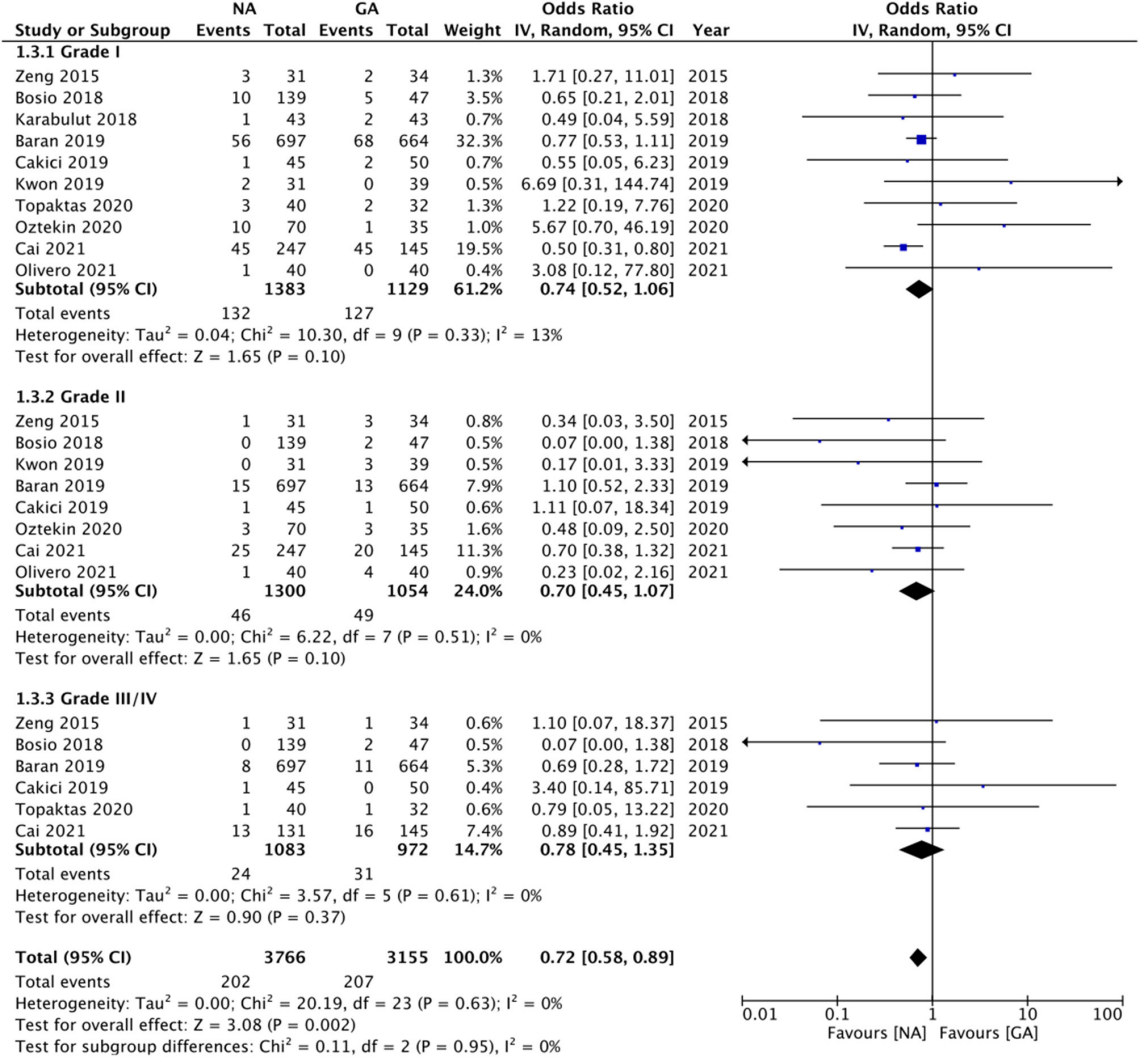
Meta-analysis of complication rates between NA and GA for RIRS.

Comparing Clavien-Dindo grade I complications, we noted no statistically significant difference between NA and GA groups (OR: 0.74 95% CI: 0.52, 1.06 I^2^ = 13% *p* = 0.10; Figure 5). On sensitivity analysis, exclusion of the studies of Kwon et al. (22) (OR: 0.70 95% CI: 0.53, 0.94 I^2^ = 3% *p* = 0.02) and Oztekin et al. (25) (OR: 0.69 95% CI: 0.52, 0.90 I^2^ = 0% *p* = 0.007) changed the significance of the results demonstrating reduced complications with NA. Subgroup analysis based on study type revealed no difference in complications between the two groups for RCTs but reduced risk of complications with NA in non-RCTs. However, there was no change in the significance of results on subgroup analysis based on NA type. The certainty of the evidence of RCTs based on GRADE was “moderate” (Supplementary Table 2).

Our results also revealed no statistically significant difference between NA and GA for Clavien-Dindo grade II (OR: 0.70 95% CI: 0.45, 1.07 I^2^ = 0% *p* = 0.10) and grade III/IV complications (OR: 0.78 95% CI: 0.45, 1.35 I^2^ = 0% *p* = 0.37; Figure 5). For grade II complications, exclusion of the study of Baran et al. (17) revealed reduced complications with NA as compared to GA (OR: 0.56 95% CI: 0.33, 0.94 I^2^ = 0% *p* = 0.03). However, the analysis of grade III/IV complications was stable on sensitivity analysis. Sub-group analyses based on study type and NA type also did not change the significance of the results (Table 2). The certainty of the evidence of RCTs based on GRADE was “low to moderate” (Supplementary Table 2). Overall, the combined data of all complications indicated a reduced risk of complications with NA as compared to GA (OR: 0.72 95% CI: 0.58, 0.69 I^2^ = 0% *p* = 0.002).

**Figure 6:**
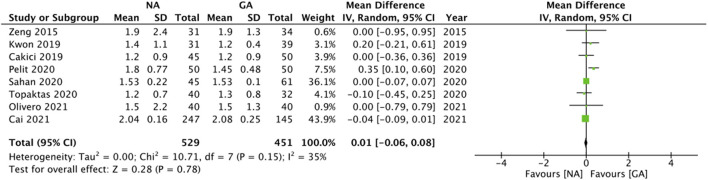
Meta-analysis of LOS between NA and GA for RIRS.

Eight studies reported data on LOS. Pooled analysis revealed no statistically significant difference between the two groups (MD: 0.01 95% CI: −0.06, 0.08 I^2^ = 35% *p* = 0.78; Figure 6). On sensitivity analysis, there was no change in the significance of the results on the exclusion of any study. Subgroup analyses also did not change the significance of the results (Table 2). The certainty of the evidence of RCTs based on GRADE was “moderate” (Supplementary Table 2).

### Risk of Bias Analysis

Details of risk of bias analysis for RCTs and non-RCTs are presented in Tables 3, 4, respectively. All RCTs were marked with a high risk of bias for non-blinding of outcome assessment, hence the overall risk of bias was also high. For non-RCTs, there was a high risk of bias in majority studies for lack of adjustment of confounding factors and for blinding of outcome assessment.

**Table 3:** Risk of bias in included RCTs.

**References**	**Randomization process**	**Deviation from intended intervention**	**Missing outcome data**	**Measurement of outcomes**	**Selection of reported result**	**Overall risk of bias**
Baran et al. (17)	Low risk	Low risk	Some concerns	High risk	Low risk	High risk
Karabulut et al. (21)	Low risk	Low risk	Low risk	High risk	Low risk	High risk
Olivero et al. (24)	Low risk	Low risk	Some concerns	High risk	Low risk	High risk
Li et al. (23)	Low risk	Low risk	Some concerns	High risk	Low risk	High risk
Oztekin et al. (25)	Low risk	Low risk	Low risk	High risk	Low risk	High risk
Topaktaş et al. (28)	Some concerns	Low risk	Low risk	High risk	Low risk	High risk
Zeng et al. (29)	Low risk	Low risk	Low risk	High risk	Low risk	High risk
Cai et al. (19)	Low risk	Low risk	Low risk	High risk	Low risk	High risk

**Table 4:** Risk of bias in included non-RCTs.

**References**	**Selection of participants**	**Confounding variables**	**Intervention measurements**	**Blinding of outcome assessment**	**Incomplete outcome data**	**Selective outcome reporting**
Bosio et al. (18)	Low risk	Low risk	Low risk	High risk	Low risk	Low risk
Baran et al. (17)	Low risk	High risk	Low risk	High risk	Low risk	Low risk
Çakici et al. (20)	Low risk	High risk	Low risk	High risk	Low risk	Low risk
Kwon et al. (22)	Low risk	High risk	Low risk	High risk	Low risk	Low risk
Sahan et al. (27)	Low risk	Low risk	Low risk	High risk	Low risk	Low risk

## Discussion

The European guidelines on the management of urolithiasis recommend the use of GA for RIRS (30). Indeed, performing the procedure under GA has advantages like increased patient comfort and better respiratory control. However, on the downside, GA is also associated with several adverse events like pulmonary complications, the possibility of drug allergies, postoperative nausea and vomiting, and very rarely, neurological and cardiac complications (31). Furthermore, patients with medical comorbidities are frequently declared unfit for GA necessitating alternate means of anesthesia for performing RIRS in such individuals. Another important factor to consider is the cost. GA results in approximately 10% higher healthcare expenditure as compared to regional anesthesia in the USA while it may be up to four times costlier in developing nations (32, 33). Surgery under GA also demands adequate infrastructure and equipment which may not be always available. In resource-limited countries of Sub-Saharan African and Southeast Asia, the use of regional anesthesia is beneficial as it provides access to healthcare in a cost-effective and secure environment (34). Considering the high prevalence of urolithiasis in the global population and the need for a safe and minimally invasive procedure to manage the disease, the feasibility of RIRS under a regional anesthetic technique like NA needs to be explored.

Our review compared outcomes of RIRS performed under NA or GA by pooling data from 13 studies thereby presenting significantly updated evidence as compared to the prior reviews (12, 13) which could include just six studies. In the analysis of our primary outcome, with data from 2,912 patients, we noted that stone-free status did not differ after surgery under NA or GA. Success rates in the NA and GA group were 80.2 and 82.9%, respectively. In comparison, the meta-analyses of Wang et al. (13) (OR: 1.07 95% CI: 0.82, 1.38) and Luo et al. (12) (OR: 0.96 95% CI: 0.91, 1.02) with a total sample of 1,747 and 580 patients, respectively have also reported similar results. Researchers have suggested that the efficacy of RIRS depends on several factors like the urinary tract anatomy, the experience of the operator, and intraoperative breathing control. Movement of the kidney and ureters during RIRS results in oscillating movements which may interfere with the precision required for laser disintegration of the stones (17). Such movements can decrease the efficacy of the procedure and increase the operative time. In this context, the use of GA can be beneficial as the anesthetist can suspend machine-controlled ventilation and control the tidal volume and breathing dynamics manually, thereby reducing visceral movements and providing a stable environment to the surgeon (35). However, the consistent results of ours and previous reviews (12, 13) indicate that such visceral movements have little impact on stone-free rates. Movements can also be restricted under NA by asking the patients to hold their breaths which can facilitate stone disintegration (21). However, it should be noted that the definition of stone-free status was heterogeneous amongst the included studies with the acceptable cut-off size of remnant stones ranging from 1 to 4 mm. Furthermore, there were variations in the postoperative imaging modality and follow-up time. Considering these differences, further studies using homogenous definitions are needed to obtain better evidence.

Similar to stone-free rates, our analysis also demonstrated no difference between NA and GA for operation time. Our results differ from the review of Wang et al. (13) which reported reduced operating time with NA but are similar to the results of Luo et al. (12) which noted no such difference. Indeed, operation time with RIRS can depend on several variables. In a recent study, Katafigiotis et al. (36) have shown that stone number, dimension, density, the type of instrument, surgeon experience, type of operating room, and use of prior nephrostomy tube are independent factors that can influence operating time with RIRS. Considering a wide-ranging of factors influencing operating time, it was not surprising to note the high heterogeneity in the meta-analysis which persisted even after subgroup analyses.

Postoperative pain is an important variable with determines patient satisfaction after a surgical procedure. In our meta-analysis, we could pool data of only 24-h pain scores between NA and GA for RIRS, which demonstrated no statistically significant difference. However, a better comparison of analgesic outcomes would have been achieved by comparing VAS scores at different intervals within the first 24 h along with the comparison of the need for rescues analgesics. Nevertheless, the heterogenous analgesic protocol amongst the included studies along with variable data reporting precluded such an analysis. In comparison, studies on other urological procedures have demonstrated that regional anesthesia offers adequate pain control as compared to GA. Tyritzis et al. (37) have shown that spinal anesthesia offers better pain control in the first 2 h after surgery while GA prevails at later stages in patients undergoing transurethral procedures. A meta-analysis comparing regional anesthesia with GA for patients undergoing PCNL has shown that regional anesthesia results in lower pain scores and reduced analgesic requirement (38).

Along with pain, another vital aspect of patient satisfaction is the incidence of complications. While GA has its own set of systemic side effects, NA can lead to complications like hypotension and headaches. However, the risk of such complications differs with different NA techniques. Spinal anesthesia has a high incidence of hypotension and headaches due to dural perforation but is easier to administer and economically reasonable. On the other hand, epidural anesthesia does not cause headaches, has a low incidence of hypotension but is comparatively difficult to administer, and can lead to insufficient blocks (20). A combination of epidural and spinal has become popular to overcome the difficulties of individual techniques. Due to inadequate data, this review could not compare the risk of specific complications between NA and GA and the risk of complications based on Clavien-Dindo grade were analyzed. On pooled analysis, we found no difference in the risk of grade I, II, and III/IV complications with the use of NA or GA for RIRS. However, considering the 95% CI of ORs, there was a small tendency of reduced risk of complications with NA as compared to GA. Also, the overall combined results indicated a 28% reduced risk of complications with NA. The results of the combined results should be interpreted with caution as they included a wide variety of complications with different study types and may not be prone to bias.

The inclusion criteria of our review were broad to include both RCTs and observational studies to present up-to-date and comprehensive evidence on the subject. However, it is well-known that observational studies are prone to selection bias. Specifically, it is plausible that patients with less complex anatomy and easily retrievable stones would have been offered NA while difficult cases would be have been carried out under GA. Furthermore, baseline differences in patient and stone characteristics would have skewed the outcomes. One method to overcome these differences is the use of propensity score matching, which, however, was carried out only by one study (24). We, therefore, segregated the current evidence based on the study type for all outcomes. Except for grade I complications, we noted that the results did not differ between RCTs and non-RCTs, which significantly adds to the credibility of our results. Secondly, the included studies also differed in the type of NA with some using combined epidural and spinal while others using only spinal anesthesia. However, in our subgroup analysis, we noted no difference in outcomes with either technique. Since the number of studies in the subgroup analyses was less, future trials should compare outcomes of RIRS under epidural vs. spinal vs. combined epidural and spinal anesthesia.

There are other limitations to our review as well. Firstly, the quality of included studies was not high for both RCTs and non-RCTs. The certainty of the evidence of RCTs was classified as “moderate” for most outcomes. Importantly there was a lack of blinding of outcome assessment in all studies which could have influenced the results. Secondly, since the studies were carried out at different centers, there would have been variations in the anesthesia and postoperative analgesia protocols. Furthermore, the influence of surgeon experience on the study outcomes cannot be negated. It may have been plausible that only experienced surgeons may have performed the procedure in some centers while a mix of novice and experienced surgeons may have been involved in other studies. Surgeon experience is an important factor influencing success rates of RIRS and breathing movements could significantly increase the risk of complications for inexperienced operators. Thirdly, we were unable to compare the incidence of specific complications like nausea/vomiting, headaches, urinary retention, etc due to limited data from the included studies. Fourthly, while the total number of studies in the meta-analysis was 13, the overall number of RCTs and non-RCTs were less than 10 and hence funnel plots may not have been appropriate to assess publication bias. Lastly, most of the included studies were from just three countries (Turkey, China, and Italy). This limits the generalizability of our findings.

## Data Availability Statement

The original contributions presented in the study are included in the article/Supplementary Materials, further inquiries can be directed to the corresponding author.

## Author Contributions

MD and YC designed the project and prepare the manuscript. MD, YC, and LS were involved in data collection and data analysis. LS edit the manuscript. All authors have read and approved the final manuscript.

## Conflict of Interest

The authors declare that the research was conducted in the absence of any commercial or financial relationships that could be construed as a potential conflict of interest.

## Publisher's Note

All claims expressed in this article are solely those of the authors and do not necessarily represent those of their affiliated organizations, or those of the publisher, the editors and the reviewers. Any product that may be evaluated in this article, or claim that may be made by its manufacturer, is not guaranteed or endorsed by the publisher.

## Supplementary Material

Supplementary Figure 1 Funnel plot for the meta-analysis of stone free rates between NA and GA.

Supplementary Table 1 Search strategy.

Supplementary Table 2 GRADE assessment of evidence.
