# Supplementary figures and images for: Outcomes of Retrograde Intrarenal Surgery Performed Under Neuraxial vs. General Anesthesia: An Updated Systematic Review and Meta-Analysis
Source: Front Surg. 2022 Mar 10;9:853875. doi: 10.3389/fsurg.2022.853875 (PMC8960175; doi:10.3389/fsurg.2022.853875)

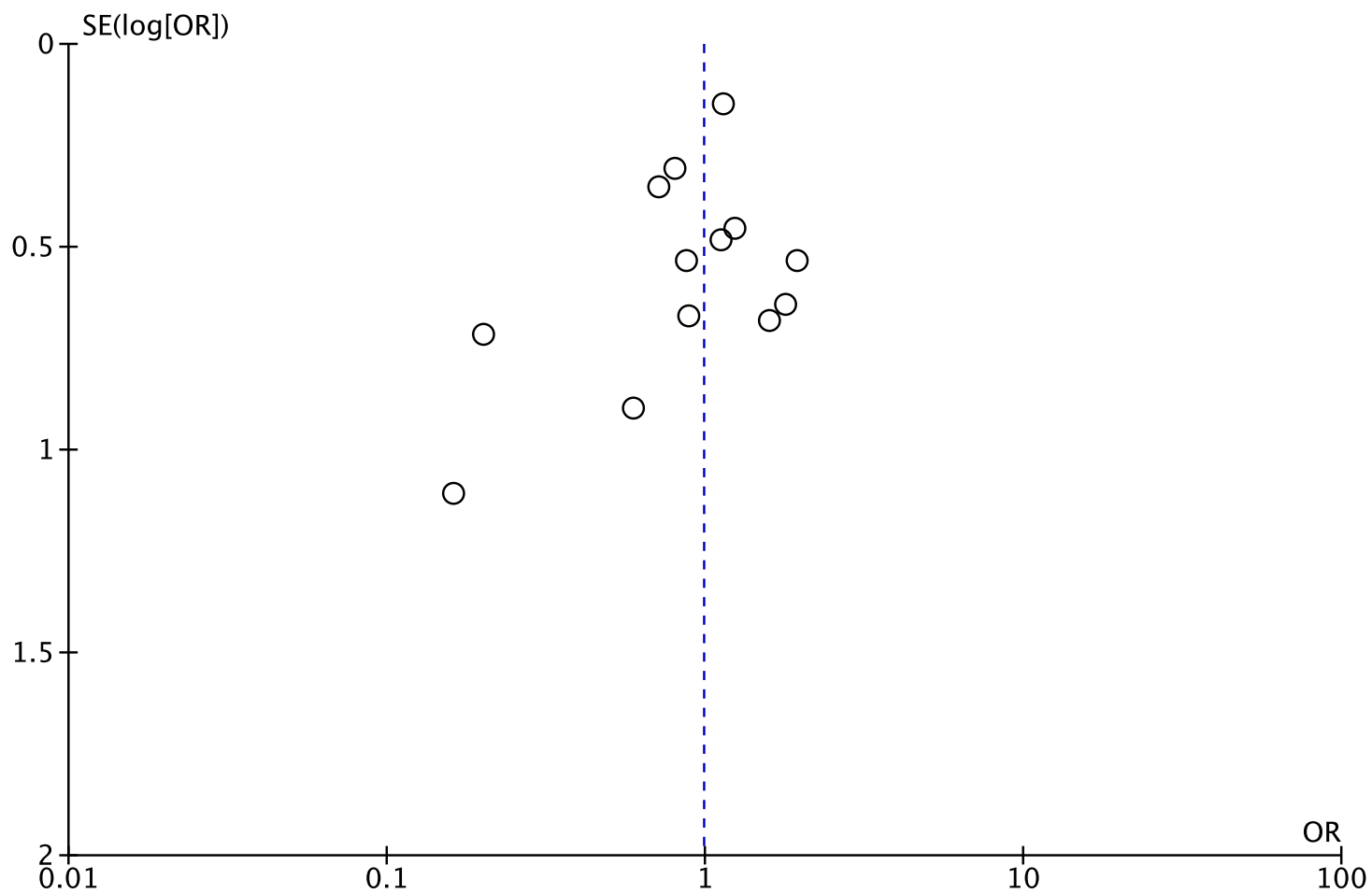

Supplement: Supplementary Figure 1 — Funnel plot for the meta-analysis of stone free rates between NA and GA. [file Data_Sheet_1.PDF]
