# Supplementary material for: Outcomes of Retrograde Intrarenal Surgery Performed Under Neuraxial vs. General Anesthesia: An Updated Systematic Review and Meta-Analysis
Source: Front Surg. 2022 Mar 10;9:853875. doi: 10.3389/fsurg.2022.853875 (PMC8960175; doi:10.3389/fsurg.2022.853875)
Supplement: Supplementary Table 1 — Search strategy. [file Table_1.DOCX]

Supplementary Table 1: Search strategy

| **Query** | **Search Details** |
| --- | --- |
| (retrograde intrarenal surgery) AND (anesthesia) | ("retrograde"[All Fields] OR "retrogradely"[All Fields]) AND ("intrarenal"[All Fields] OR "intrarenally"[All Fields]) AND ("surgery"[MeSH Subheading] OR "surgery"[All Fields] OR "surgical procedures, operative"[MeSH Terms] OR ("surgical"[All Fields] AND "procedures"[All Fields] AND "operative"[All Fields]) OR "operative surgical procedures"[All Fields] OR "general surgery"[MeSH Terms] OR ("general"[All Fields] AND "surgery"[All Fields]) OR "general surgery"[All Fields] OR "surgery s"[All Fields] OR "surgerys"[All Fields] OR "surgeries"[All Fields]) AND ("anaesthesia"[All Fields] OR "anesthesia"[MeSH Terms] OR "anesthesia"[All Fields] OR "anaesthesias"[All Fields] OR "anesthesias"[All Fields]) |
| (RIRS) AND (anesthesia) | "RIRS"[All Fields] AND ("anaesthesia"[All Fields] OR "anesthesia"[MeSH Terms] OR "anesthesia"[All Fields] OR "anaesthesias"[All Fields] OR "anesthesias"[All Fields]) |
| (ureterolithotripsy) AND (anesthesia) | "ureterolithotripsy"[All Fields] AND ("anaesthesia"[All Fields] OR "anesthesia"[MeSH Terms] OR "anesthesia"[All Fields] OR "anaesthesias"[All Fields] OR "anesthesias"[All Fields]) |
| (FURS) AND (anesthesia) | "FURS"[All Fields] AND ("anaesthesia"[All Fields] OR "anesthesia"[MeSH Terms] OR "anesthesia"[All Fields] OR "anaesthesias"[All Fields] OR "anesthesias"[All Fields]) |
| (ureteroscopy) AND (anesthesia) | ("ureteroscopy"[MeSH Terms] OR "ureteroscopy"[All Fields] OR "ureteroscopies"[All Fields]) AND ("anaesthesia"[All Fields] OR "anesthesia"[MeSH Terms] OR "anesthesia"[All Fields] OR "anaesthesias"[All Fields] OR "anesthesias"[All Fields]) |
