# Supplementary material for: Outcomes of Retrograde Intrarenal Surgery Performed Under Neuraxial vs. General Anesthesia: An Updated Systematic Review and Meta-Analysis
Source: Front Surg. 2022 Mar 10;9:853875. doi: 10.3389/fsurg.2022.853875 (PMC8960175; doi:10.3389/fsurg.2022.853875)
Supplement: Supplementary Table 2 — GRADE assessment of evidence. [file Table_2.DOCX]

| **Supplementary Table 2: GRADE assessment of evidence** | | | | | | | | | | | |
| --- | --- | --- | --- | --- | --- | --- | --- | --- | --- | --- | --- |
| **Certainty assessment** | | | | | | | **Summary of findings** | | | | |
| **Participants (studies) Follow-up** | **Risk of bias** | **Inconsistency** | **Indirectness** | **Imprecision** | **Publication bias** | **Overall certainty of evidence** | **Study event rates (%)** | | **Relative effect (95% CI)** | **Anticipated absolute effects** | |
|  |  |  |  |  |  |  | **With General anesthesia** | **With Neuraxial anesthesia** |  | **Risk with General anesthesia** | **Risk difference with Neuraxial anesthesia** |
| **Stone free rate** | | | | | | | | | | | |
| 821 (8 RCTs) | serious^a^ | not serious | not serious | not serious | none | ⨁⨁⨁◯ Moderate | 359/417 (86.1%) | 347/404 (85.9%) | **OR 0.97** (0.56 to 1.65) | 861 per 1,000 | **4 fewer per 1,000** (from 85 fewer to 50 more) |
| **Operation time** | | | | | | | | | | | |
| 735 (7 RCTs) | serious^a^ | not serious | not serious | not serious | none | ⨁⨁⨁◯ Moderate | 374 | 361 | - | The mean operation time was **0** | MD **1.33 higher** (6.66 lower to 9.31 higher) |
| **Pain 24 hrs** | | | | | | | | | | | |
| 446 (5 RCTs) | serious^a^ | not serious | not serious | not serious | none | ⨁⨁⨁◯ Moderate | 219 | 227 | - | The mean pain 24 hrs was **0** | MD **0.07 lower** (0.41 lower to 0.26 higher) |
| **Complications** | | | | | | | | | | | |
| 916 (5 RCTs) | serious^a^ | not serious | not serious | not serious | none | ⨁⨁⨁◯ Moderate | 18/443 (4.1%) | 24/473 (5.1%) | **OR 1.00** (0.49 to 2.04) | 41 per 1,000 | **0 fewer per 1,000** (from 20 fewer to 39 more) |
| **Complications - Grade I** | | | | | | | | | | | |
| 421 (5 RCTs) | serious^a^ | not serious | not serious | not serious | none | ⨁⨁⨁◯ Moderate | 7/201 (3.5%) | 17/220 (7.7%) | **OR 1.73** (0.62 to 4.82) | 35 per 1,000 | **24 more per 1,000** (from 13 fewer to 113 more) |
| **Complications - Grade II** | | | | | | | | | | | |
| 335 (4 RCTs) | serious^a^ | not serious | not serious | not serious | none | ⨁⨁⨁◯ Moderate | 10/158 (6.3%) | 5/177 (2.8%) | **OR 0.44** (0.14 to 1.35) | 63 per 1,000 | **34 fewer per 1,000** (from 54 fewer to 20 more) |
| **Complications - Grade III/IV** | | | | | | | | | | | |
| 160 (2 RCTs) | serious^a^ | not serious | not serious | serious^b^ | none | ⨁⨁◯◯ Low | 1/84 (1.2%) | 2/76 (2.6%) | **OR 1.79** (0.21 to 14.96) | 12 per 1,000 | **9 more per 1,000** (from 9 fewer to 141 more) |
| **LOS** | | | | | | | | | | | |
| 436 (5 RCTs) | serious^a^ | not serious | not serious | not serious | none | ⨁⨁⨁◯ Moderate | 234 | 202 | - | The mean LOS was **0** | MD **0.11 higher** (0.06 lower to 0.29 higher) |

**CI:** confidence interval; **MD:** mean difference; **OR:** odds ratio; **LOS**: Length of hospital stay

#### Explanations

a. Overall "high risk" of bias in all included studies

b. limited number of studies
